# Two-Component Systems of *S. aureus*: Signaling and Sensing Mechanisms

**DOI:** 10.3390/genes13010034

**Published:** 2021-12-23

**Authors:** Lisa Bleul, Patrice Francois, Christiane Wolz

**Affiliations:** 1 Interfaculty Institute of Microbiology and Infection Medicine, University of Tübingen, 72076 Tubingen, Germany; Lisa.bleul@med.uni-tuebingen.de; 2 Cluster of Excellence EXC 2124 “Controlling Microbes to Fight Infections”, University of Tübingen, Elfriede-Aulhorn-Str. 6, 72076 Tubingen, Germany; 3 Genomic Research Laboratory, Infectious Diseases Service, University Hospitals of Geneva University Medical Center, Michel Servet 1, CH-1211 Geneva, Switzerland; Patrice.Francois@unige.ch

**Keywords:** *S. aureus*, two-component systems, histidine kinase, signal sensing, ligand

## Abstract

*Staphylococcus aureus* encodes 16 two-component systems (TCSs) that enable the bacteria to sense and respond to changing environmental conditions. Considering the function of these TCSs in bacterial survival and their potential role as drug targets, it is important to understand the exact mechanisms underlying signal perception. The differences between the sensing of appropriate signals and the transcriptional activation of the TCS system are often not well described, and the signaling mechanisms are only partially understood. Here, we review present insights into which signals are sensed by histidine kinases in *S. aureus* to promote appropriate gene expression in response to diverse environmental challenges.

## 1. Introduction

The human bacterial pathogen *S. aureus* asymptomatically colonizes approximately 20% of the general population [1]. However, *S. aureus* is also a major human pathogen that causes a variety of acute and chronic diseases [2,3]. The versatility of this organism arises due to its capacity to produce accessory molecules that mediate specific interactions with host cells and to quickly adapt to changing environments. Gene expression is tightly controlled by an interactive regulatory network. Two-component systems (TCSs) are central pillars of environmental sensing [4,5]. *S. aureus* encodes 16 TCSs that are evenly distributed on the bacterial chromosome, and some methicillin-resistant *S. aureus* (MRSA) encode one additional TCS on an accessory SCC-*mec* island [6]. Among the 16 systems, only WalRK is essential for bacterial growth, whereas the others can be deleted simultaneously in the same strain without affecting cell viability under laboratory conditions [6].

However, most TCSs contribute to better survival or modulation of virulence under certain infectious conditions [4,7]. Each TCS is mostly autonomous in sensing and responding to its cognate signals, with limited cross-regulation between the systems [6]. In many cases, the signals leading to TCS activation and the underlying molecular mechanisms are not well understood. In terms of function, a canonical TCS consists of a histidine kinase (HK) that is autophosphorylated at a conserved histidine residue and then transfers the phosphoryl group to an aspartate residue in the cognate response regulator (RR) [8]. Many HKs also elicit phosphatase activity, which is important for resetting the system [9]. HKs are usually membrane-bound homodimers that receive signals via defined transmembrane, extracellular, or intracellular sensing domains [10,11]. The cytoplasmic C-terminus includes a linker or HAMP domain (not always present), additional sensory domains, a conserved dimerization and histidine-containing domain (DHp), and a catalytic ATP binding (CA) domain. After sensing a signal, a conformational change generally occurs in the sensor portion of the protein that triggers the autophosphorylation of the conserved moieties of the protein [12].

**Figure 1:**
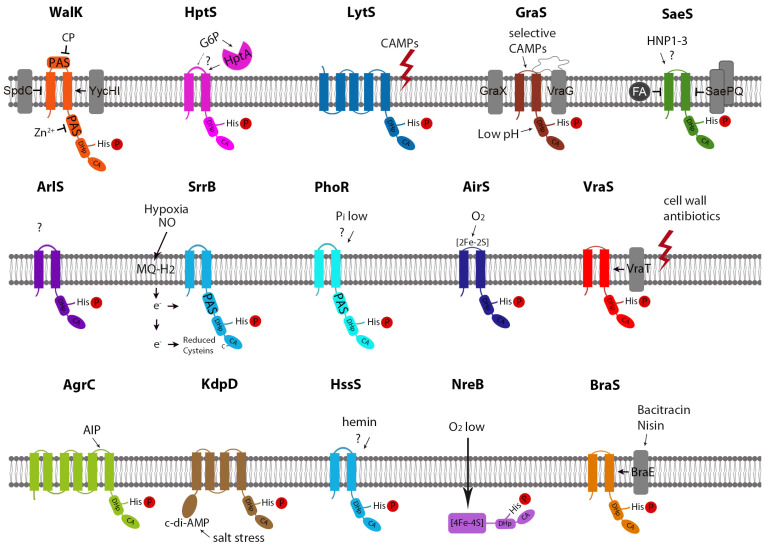
Structure and Signaling-Mechanisms of *S. aureus* HKs. CP: Peptidoglycan cleavage products; CAMP: Cationic antimicrobial peptide; FA: fatty acid.

In *S. aureus*, four HKs are classified as intramembrane-sensing histidine kinases (IM-HKs), namely, SaeS, VraS, GraS, and BraS [13]. One HK, AgrB, is a quorum sensor that enables the bacteria to monitor cell density [4,14]. PhoR and WalK are the only HKs in *S. aureus* that harbors a Per-ARNT-Sim (PAS) domain as a potential ligand interaction site [15]. Two *S. aureus* HKs, namely, AirS and NreB, are iron-sulfur (Fe-S) cluster-containing proteins (Figure 1).

Many TCS gene clusters contain additional proteins, some of which are known to interact with the function of the TCS (Table 1). The TCS-encoding operons are often autoregulated, providing a positive feedback loop, or are under the control of other regulators. However, an increase in TCS expression does not necessarily result in an increase in target gene expression, e.g., under conditions when the native signal for HK activation is missing. Moreover, overexpression may also result in inhibition through the coactivation of accessory genes with inhibitory function (e.g., *saePQ*). Thus, the gene expression levels of TCSs are often not informative for identifying cognate signals, and analyses of defined reporter genes might be more suitable. In general, operon architecture and overall functions are well studied by phenotypic and genetic analyses of mutant strains. Target genes are often localized next to the TCSs but can also be scattered throughout the chromosome. The dedicated signals and the mechanism by which TCSs sense their signals are still largely unknown. Here, we will summarize recent knowledge on the signaling mechanism of individual *S. aureus* HKs.

## 2. WalRK

The WalRK (also called YycGF) system was first described in *Bacillus subtilis* [16,17] and is highly conserved in low G+C Gram-positive bacteria [18,19]. In *S. aureus*, WalRK regulates the expression of genes involved in cell wall metabolism, thereby controlling autolysis, biofilm formation, and virulence [20,21,22]. WalRK function is strongly associated with the vancomycin-intermediate resistant *S. aureus* (VISA) phenotype. Clinical VISA strains, as well as resistant strains selected in vitro, often contain single nucleotide polymorphisms (SNPs) within the WalRK system [23,24,25]. The HK WalK is encoded within a 4-gene operon (*walRKHI*), which seems not to be autoregulated. WalK from *S. aureus* harbors an intracellular and an extracellular PAS domain presumably involved in sensing [26]. The cytoplasmic PAS domain in *B. subtilis* [27], and presumably also that in *S. aureus* [28], is essential to localize WalK to the division septum. This domain (amino acids: from 261 to 375) is conserved among members of *Staphylococcus* species and contains four critical and highly conserved residues (His 271, Asp 274, His 364, and Glu 368) that are involved in Zn^2+^ binding [29]. The WalKH271Y mutation abrogates metal binding, increasing WalK kinase activity and WalR phosphorylation. Thus, Zn^2+^ binding to WalK is important to restrict WalK activity. Recently, an altered WalK-H364 residue within the Zn^2+^ binding region, detected in VISA strains, was predicted to destabilize the protein and decrease WalK activity [30]. Interestingly, another VISA-related WalK mutation (Y306N) located in the cytoplasmic PAS domain increased WalK kinase activity in liposomes [31].

Deletion of the extracellular PAS domain of *B. subtilis* WalK results in constitutive signaling [27,32]. In this organism, peptidoglycan cleavage products generated by D,L-endopeptidase digestion specifically inhibit WalK activity, presumably by interaction with the extracellular PAS domain [32]. It is likely that the homologous PAS domain of *S. aureus* WalK also senses peptidoglycan degradation products. This would support a model in which WalK is inhibited when it binds to its signal and activated when unliganded.

WalK was previously proposed to be involved in sensing the D-Ala-D-Ala moiety of Lipid II as a signal for active cell wall synthesis [19]. This conclusion was mainly based on the observation that different cell wall-targeting antibiotics affect the WalRK regulon in *B. subtilis.* Recent studies suggest that this finding is likely mediated by serine/threonine kinase PknB activity. The interaction of Lipid II with PknB stimulates the phosphorylation of the RR WalR [33]. Amino acid T101, which represents the sole PknB phosphorylation site on WalR, is located in the vicinity of the dimerization interface of the regulator and might therefore influence dimerization and DNA binding behavior.

The genes that encode two additional membrane proteins, *yycH* and *yycI*, are co-transcribed with *walRK*, and the gene products modulate WalK activity. In contrast to *B. subtilis*, the disruption of the *yycH* and *yycI* genes in *S. aureus* led to a downregulation of the WalRK regulon [34]. Both proteins were predicted to interact with WalK in a bacterial two-hybrid system, most likely forming a ternary complex via their transmembrane domains [34]. Phosphorylation assays with full-length recombinant proteins in phospholipid liposomes confirmed that YycH and YycI stimulate WalK activity [35]. Recently, SpdC, an Abi (abortive infectivity)-domain membrane protein, was described to also interact with WalK at the division septum and to negatively control the expression of WalRK regulon genes [36].

Thus far, the main modulators of WalK activity (Zn^2+^, peptidoglycan cleavage products, SpdC) inhibit WalK. The question remains whether there is any true signal that activates WalK (possible acting via YycHI) or whether regulation primarily occurs via inhibition of an otherwise constitutively active (kinase-On) WalK system.

## 3. HptASR

The HptASR (hexose phosphate transport) system regulates glucose-6-phosphate (G6P) uptake by activating the hexose phosphate antiporter UhpT [37,38]. The system appears to be important for intracellular survival because the carbon source in the cytoplasm of host cells is limited to hexose phosphate. The *hptASR* mutant is impaired in survival/multiplication within various types of host cells and gives resistance to fosfomycin [37]. *uhpT* is also differentially expressed, e.g., in biofilm-forming bacteria, during acidification or oxidative stress, which likely involves control by the catabolite control protein A (CcpA) system. CcpA represses *hptSR* in a glucose-dependent manner, thereby coordinating G6P uptake and metabolism when glucose is limited [39].

G6P sensing of the HptASR system is mediated by HptA, a secreted protein that binds to G6P directly and then transfers the signal to the membrane-bound HK HptS. The solved structures of HptA in complex with the periplasmic domain of HptS showed that HptA forms a tetramer with HptS [40]. The G6P-free form of HptA binds to the distal membrane side of the HptS periplasmic domain. G6P binding to HptA switches the contact region to the membrane-proximal domain of HptS. This causes untwisting and tilting movement in the TM2 region of HptS. This conformational change is then transmitted to the HAMP domain to allow kinase activation. However, G6P might also directly interfere with HptS because a strain lacking HptA is completely abolished G6P sensing [38].

## 4. LytSR

The LytSR system was identified as a TCS that affects autolysis in *S. aureus* through the activation of the *lrgAB* operon [41]. It was proposed that LrgA, together with the homologous CidA, functions as a holin/anti-holin system to regulate murein hydrolase activity and, ultimately, cell death. *lytSR* expression is positively modulated by the direct binding of SpoVG to a short consensus sequence within the 5’ noncoding region of *lytSR* [42]. The HK LytS harbors five transmembrane regions that are likely involved in sensing alterations in the membrane potential and thereby acting as a “voltmeter” [43,44]. His390 of LytS is the site of autophosphorylation, and Asn394 is a critical amino acid involved in phosphatase activity [45]. Cationic antimicrobial peptides (CAMPs) usually alter membrane potential; therefore, LytS might function as a universal CAMP sensor and responder. Consistent with this assumption, *lytSR* mutants were shown to be more sensitive to CAMP [43]. This makes the system important for in vivo survival because it enables a general response to CAMPs in contrast to the rather selective response mediated by the other CAMP-sensing TCSs, namely, GraRS and SaeRS.

Of note, under specific conditions, acetyl phosphate can act as phosphodonor for the RR LytR, thereby bypassing LytS [45,46].

## 5. GraRS

The GraRS system, also called ApsSR [47], mediates resistance to several CAMPs and vancomycin mainly via regulation of *vraFG*, *mprF*, and *dltABCD* [47,48,49,50]. The regulon was shown to be activated by indolicidin, mellitin, nisin, LL-37 [47], colistin [50], RP-1, and polymyxin B (PMB) but not by HNP-1, vancomycin, gentamycin, or daptomycin [51]. GraS belongs to the subset of IM-HK, in which two transmembrane helices frame a very short extracellular loop (EL) [13]. For the homologous GraS/ApsS HK of *S. epidermidis,* the predicted 9-amino-acid EL, which harbors a high density of negative charges, directly binds to CAMPs [52]. The ELs of GraS/ApsS of *S. aureus* and *S. epidermidis* show 33% similarity, resulting in different degrees of selectivity for CAMPs [47]. Direct interaction of the EL loop region (DYDFPIDSL) with CAMPs is also likely in *S. aureus*, since a soluble EL mimic of GraS protected *S. aureus* against CAMP-mediated death [53]. The proline at position 39 and the two aspartic acid residues at positions 37 and 41 located within the EL are important for full GraS activity [53,54,55].

GraRS is part of a three-gene operon (*graXRS*) localized immediately upstream of the ABC transporter genes *vraF* and *vraG* [56]. Whereas the *graXRS* operon does not seem to be autoregulated, *vraFG* expression is under the tight control of GraRS [56]. The auxiliary factor GraX is required for colistin-mediated activation of the system [56] and likely functions as a scaffold to promote protein interactions with GraS, GraR, and VraFG [57].

The GraR target gene *vraG* encodes a membrane permease, while *vraF* encodes an ATPase to provide energy for efflux. Interestingly, a *vraG* mutant phenocopied a *graS* mutant with respect to CAMP sensing [58]. Native VraG interacted with GraS to restrict its kinase activity [58]. VraG contains a single 200-residue EL located between the seventh and eighth transmembrane segments. Deletion of the VraG-EL domain reduced the interaction and resulted in GraS activation even without CAMPs. It is hypothesized that the tight interaction between VraG-EL and GraS-EL is weakened by the binding of CAMPs, resulting in the activation of GraS. Specific lysine residue(s) on the VraG-EL presumably reduces the sensing of cognate CAMPs by GraS-EL. Thus, VraG, more than an efflux pump, facilitates GraS-mediated sensing of HDPs [58].

Recent work has expanded the sensory capabilities of GraS to include the sensing of acidic pH [54,55,59], and GraS activation at low pH is mandatory for survival within macrophages [58]. Interestingly, pH-dependent modulation of GraS activity seems to be independent of the Gra-EL implicated in CAMP sensing [58]. Acidic pH reduces the charge on polar lipid head groups, which presumably impacts GraS activity. It was hypothesized that acidic pH should also reduce the charge on acidic amino acids in the EL of GraS, which would render them less effective in sensing CAMP at low pH [55]. An EL-independent mechanism of GraS activation under acidic conditions might be beneficial for the bacterium. Thus, low pH and CAMPs present during infection seems to independently signal through GraS to promote bacterial survival.

## 6. SaeRS

The SaeRS system first described by Giraudo et al. [60] regulates the expression of numerous virulence factors in *S. aureus*, including surface-bound and secreted proteins [60,61,62,63]; for a comprehensive review about the organization of the *sae* operon, consensus sequences, and regulated target genes, see [64]. The Sae system was shown to be induced by phagocytosis-related signals, of which human neutrophil peptides 1–3 (HNP1–3) showed the most pronounced effect [65]. SaeS activation by subinhibitory concentrations of HNP1–3 was confirmed in strains of the USA300 lineage [66,67]. However, the widely used strains 8325 and Col were found to be nonresponsive to HNP1–3 sensing [65].

SaeS is coded within a four-gene operon, *saePQRS,* transcribed from two promoters (P1 and P3). The mature full-length transcript is transcribed from the autoactivated P1 promoter, and a stable RNA is generated by RnaseY-dependent endoribonucleolytic cleavage between *saeP* and *saeQ* [68]. *saePQRS* expression is decreased in *agr* mutants [65,69]. However, Agr and Sae impact virulence gene expression primarily independent of each other [64]. The P1 promoter was also shown to be under the control of the metabolic regulator CodY, and CodY also indirectly regulates *sae* expression through Agr- and Rot-mediated repression of the *sae* P1 promoter [70]. One additional constitutive P3 promoter located within *saeQ* ensures a basal level of *saeRS* expression [71,72].

HK SaeS is a IM-HK [13] with a short 9-aa EL. A single amino acid substitution in the first transmembrane helix (L18P) of the EL in the Newman strain renders the system hyperactive [73]. The overall confirmation of the transmembrane domain, as well as the EL peptide composition, are involved in signal transduction [64,66].

It was proposed that in the signal transfer process, the entire N-terminal domain of SaeS (two transmembrane helices and the EL) works as a coherent unit in a tripwire manner. In this “tripwire” model, the overall conformation of the entire N-terminal domain is the key determinant in controlling the kinase activity of HK. Any stimulus that elicits conformational changes in the N-terminal domain is expected to either repress or activate the kinase activity of the HK, depending on the nature of the conformational change [64].

This model predicts that molecules that alter the conformation of the transmembrane domain can serve as signal transducers to regulate SaeS activity. However, despite the well-documented specific effect of HNPs on SaeS activation, it is still unclear whether these peptides directly bind to SaeS or whether they act via a hitherto-unidentified receptor molecule. The latter theory is supported by the observation that not all *S. aureus* strains are sensitive to HPN despite harboring identical SaeRS alleles [65].

In addition to HPNs, additional factors can modulate SaeS activity. The cotranscribed lipoprotein SaeP and the membrane protein SaeQ are dispensable for the activation of the Sae system [73,74]; however, they are required to induce SaeS phosphatase activity. Thus, SaePQ helps to set the system to a pre-activation state via a negative feedback mechanism. The membrane protein SpdC was proposed to interact with the transmembrane domains of WalK and SaeS [36]. The silkworm apolipophorin protein binds to lipoteichoic acid (LTA), and LTA complexed with the protein inhibits the kinase activity of SaeS by interacting with the transmembrane domain of SaeS [75]. In an in vitro enzyme assay, Cu, Zn and Fe ions were shown to inhibit the kinase activity of SaeS [76], possibly by competing with Mg. Furthermore, the fatty acid kinase VfrB was proposed to positively control SaeRS-mediated transcription of virulence factors [77].

Recently, excess levels of both external and cytoplasmic free fatty acids were proposed to modulate bacterial virulence factor production via SaeRS inhibition [77,78]. Unsaturated fatty acids are generally stronger inhibitors than saturated fatty acids [79]. Accordingly, deletion of respiratory dehydrogenase genes, which leads to altered NAD^+^/NADH ratios and free fatty acid accumulation, is accompanied by reduced SaeRS activity [80]. Fatty acid sensitivity occurs independently of SaePQ but requires the native SaeS transmembrane [79]. Thus, SaeS probably integrates membrane-active signals to either increase (HPN1) or decrease (fatty acids) SaeS kinase activity.

## 7. TCS-7, DesKR

One TCS has thus far not been characterized in *S. aureus* and is thus provisionally named TCS-7. A four-gene cluster that encodes an ABC transporter system and the TCS is conserved among Firmicutes. Recently, *S. aureus* HK (SA1313) was expressed in *B. subtilis* and shown to functionally complement the *B. subtilis* homologue DesK [81]. The TM domain of *B. subtilis* DesK was shown to be essential for signal detection and modulation of its catalytic activities and thought to be involved in temperature sensing [17]. Similar to *B. subtilis* DesK, the *S. aureus* homologue adopts a kinase-ON state at low temperature and phosphorylates DesR. However, both enzymes behave differently in the context of changes in the cytoplasmic membrane properties caused by isothermal conditions. Such differences likely occur due to major differences in the TM domain. The two co-transcribed genes (SauSa300_1217 and SauSa300_1218) encode putative ABC transporter systems with unknown functions. Further analyses in *S. aureus* are required to prove that DesKR is involved in temperature sensing and determine whether and how the putative ABC transporter is functionally linked to DesKR activity.

## 8. ArlRS

The ArlRS (autolysis-related locus) system [82] regulates the expression of several virulence factors [83]. After sensing a yet unknown signal, activated ArlR, in turn, drives the expression of the global regulators MgrA [84,85] and Spx [86]. The Spx regulator controls the *S. aureus* response to ß-lactam antibiotics and stress, while the global regulator MgrA directly impacts virulence by controlling the expression of over 100 effector genes. The expression of *arlRS* and some *arl* target genes is sensitive to DNA supercoiling [87,88]. However, little is known about a potential activating signal of the HK ArlS. There is no special motif predicted in the sensor domain, but the 118-amino acid extracellular region between the two membrane-spanning domains (InterPro prediction) could be a potential ligand interaction site. Recent studies revealed that ArlS is necessary for the activation of ArlR in response to manganese starvation caused by calprotectin and glucose limitation [89,90].

## 9. SrrAB

The SrrAB (staphylococcal respiratory response) system [91] regulates the expression of genes involved in anaerobic metabolism, nitrosative stress, and cytochrome biosynthesis [92,93]. The HK SrrB responds to hypoxia and nitric oxide (NO), possibly by sensing reduced menaquinone levels as a result of impaired electron flow in the electron transport chain [92,93,94]. SrrB contains an extracellular cache domain and a cytoplasmic HAMP-PAS region [95]. The structure of the catalytic domain revealed a unique intramolecular cysteine disulfide bond in the ATP-binding domain [95], rendering the SrrB kinase activity sensitive to the cysteine redox state. It was proposed that under aerobic conditions, cysteines are oxidized to keep kinase activity low. Under anaerobic conditions, the pool of reduced menaquinones increases, resulting in reduced SrrB cysteines and higher kinase activity. However, fully oxidized SrrB retains ~60% activity, and cysteines are not conserved in all staphylococcal species [95]. Thus, additional molecular mechanisms are likely involved in modulating SrrB activity. The PAS domain itself significantly influenced SrrB kinase and phosphatase activity in vitro [96]. Redox-active ligand binding to the PAS domain may further affect SrrB activity. Moreover, the role of the extracellular Cache domain in SrrB activation remains unclear.

## 10. PhoPR

Inorganic phosphate (Pi) is an essential nutrient required in multiple cellular processes, including energy metabolism and intracellular signaling. However, this nutrient is generally found at very low concentrations in its free form and is directly utilizable by bacterial cells. The PhoPR system is essential for phosphate homeostasis in *S. aureus* by sensing phosphate limitation and regulating the expression of three phosphate transporters, namely, PstSCAB, NptA, and PitA [97]. In addition to phosphate transporter expression, PhoPR controls factors contributing to virulence. In the homologous system in *E. coli,* the PhoR kinase interacts through its PAS domain with the negative regulator PhoU, which monitors the activity of the Pi-specific ATP-binding cassette transporter PstSCAB [98]. Low activity of the Pst transporter then results in a high activity of PhoR. Signal sensing is mediated through conformational changes of PstB in response to Pi availability [99]. In *B. subtilis,* no homologue to PhoU is present, and PhoR activity is responsive to biosynthetic intermediates of wall teichoic acid (WTA) metabolism [100]. Considering that the respective WTA intermediate is not present in *S. aureus* and that PhoU is not needed for the growth of *S. aureus* in Pi-depleted medium, Pi sensing in *S. aureus* is fundamentally different from that in *E. coli* and *B. subtilis* [97]. Nevertheless, the intracellular PAS domain can be assumed to be important for ligand interactions. The functionality of the phosphate homeostasis system appears important for bacterial pathogenesis, as cells expressing mutant *phoPR* showed an altered capacity to invade multiple organs in a mouse model of infection [97]. It should be noted that this phenomenon occurs independently of the presence of the phosphate transporter NptA. This observation might be related to the involvement of Pi homeostasis in the maintenance of cell wall structure in Gram-positive bacteria, as revealed by the impact of Pi on resistance to cell wall-active antibiotics, such as glycopeptides [101].

## 11. AirSR

The AirSR (anaerobic iron-sulfur cluster-containing redox sensor regulator) system [102], also called YhcSR [103], responds to oxidation signals. AirSR contributes to resistance to H_2_O_2_, autolysis, survival in blood, and vancomycin resistance [104,105]. The N-terminal sensor domain of HK AirS contains a Fe-S cluster essential for its kinase activity [103]. Under anaerobic conditions or in the absence of ROS, the Fe-S cluster is reduced, leading to low phosphorylation of AirS. When exposed to oxygen, [2Fe-2S]^+^ is oxidized to [2Fe-2S]^2+^, which leads to full kinase activity of AirS. Overoxidation and exposure to H_2_O_2_ or NO stress results in protein inactivation. Consistent with this model, only a few genes were affected by AirRS when bacteria were grown anaerobically [105] since the reduced AirS should have low kinase activity under this condition. The regulation occurred through direct binding to the promoter region of target genes. Surprisingly, in a previous study, it was found that AirR only affects gene expression under anaerobic conditions in strain Newman [102]. This discrepancy may suggest that the regulatory activity of AirR is strain-specific. Why AirSR acts so differently in different strains remains unclear. Regarding physiopathology, this TCS has been described to contribute to *S. aureus* survival in human blood by transcriptionally regulating the expression of *sspABC*, which encodes important proteases that mediate the hydrolysis of opsonins, thus inhibiting killing by professional phagocytic cells [104].

## 12. VraSR

The VraSR (vancomycin resistance associated) system facilitates resistance to cell wall antibiotics via the regulation of cell wall biosynthesis and antibiotic resistance genes [106]. Activation of VraSR target genes is triggered by cell wall synthesis inhibitors such as glycopeptides or ß-lactam antibiotics. A similar conserved VraSR-linked cell wall stress response can be evoked by inhibition of peptidoglycan ligase [107] or penicillin-binding protein *pbpB* [108]. The HK coding *vraS* is part of an autoregulated four-gene operon (*vraUTSR*) [109]. VraS also belong to the family of IM-HK [13]. The molecular mechanism involved in VraS sensing remains unclear; however, the interaction between VraT and VraS seems to be involved. Deletion of the putative membrane protein VraT leads to constitutive *vra* regulon expression [110]. It was suggested that cell wall damage modifies the VraT-VraS interaction to influence VraS kinase activity.

## 13. AgrCA

The AgrCA (accessory gene regulator) system [111] senses population density through the production of an autoinducing peptide (AIP) and serves as a regulator of global virulence mostly via the regulatory RNAIII and the transcription factor Rot (for reviews see [4,112,113,114,115]. The system is composed of a 4-gene operon (*agrBDCA*) and the divergently transcribed regulatory RNAIII, which encodes *hld*. AIP is a thiolactone peptide (8–9 aa) that is processed from the AgrD-encoded propeptide by AgrB [116]. There are several AIP derivatives that interact with cognate AgrC to activate kinase activity. For *S. aureus,* four Agr specificity groups were identified (Groups I–IV). Additionally, AIPs inhibit noncognate AgrC. The Agr circuit is conserved in many staphylococcal species, with each producing its own unique AIP [117]. Therefore, Agr polymorphisms contribute to strain interference between staphylococci of different Agr groups. The thiolactone ring of AIP is necessary for binding to AgrC, whereas the tail is critical for activation. Deletion of the tail converts AIP from an agonist into an antagonist molecule [118]. The structural features of both native AIPs and non-native analogues were revealed using NMR spectroscopy [119,120,121]. Two critical structural motifs within the AIP-III ligand were identified: (i) a hydrophobic patch (or “knob”) on the macrocycle essential for receptor binding and (ii) an additional hydrophobic contact or “anchor” on the N-terminal tail critical for receptor activation. In the absence of the anchor, peptides containing a hydrophobic knob were found to inhibit the AgrC-III receptor, presumably by outcompeting the native ligand.

The HK AgrC is classified as a member of the ‘‘HPK10’’ kinase subfamily [122] in which an Asn residue substitutes for the conserved ‘‘G1-box’’ Asp, which normally hydrogen-bonds to the N-6 amino group on the adenine base of the nucleotide. Therefore, the Asn residue is likely responsible for the exceptionally weak affinity between full-length AgrC and ATP, which renders the kinase activity of AgrC strongly dependent upon the cellular ATP level. Thus, when energy starvation decreases the cellular ATP level, the AgrC kinase activity will be diminished even in the presence of AIP activators.

The N-terminal sensor domain spans the membrane six times. A short peptide sequence with a high helical propensity, termed the S helix, links the sensor domain with the cytoplasmic DHp and CA domains. AIPs bind to AgrC-I at 2:2 stoichiometry. The binding of agonist or inverse-agonist peptides results in twisting of the linker in different directions, thus either promoting or inhibiting kinase activity. However, AIP signals do not regulate signaling at the level of RR dephosphorylation [122].

The crystal structure of the AgrC HK module trapped in the apo form showed that the CA domain docks against the DHp helices in a manner that prevents histidine autophosphorylation [123]. Noncovalent interaction between R238 and Q305 stabilizes AgrC in the “off” state. This “latch” is proposed to lift after the binding of AIP to the extracellular AgrC sensor domain. Constitutive mutations discovered in the HK module, e.g., R238, localize to the docking interface [124]. Naturally occurring mutations in AgrC are consistent with repositioning of key functional domains of AgrC [125].

The mechanism(s) by which ligand binding induces these structural changes has yet to be resolved. It was proposed that AIP binding stabilizes or induces discrete rotational conformations in the last helix of the sensor that is directly transduced into the DHp domain by the S helix [123].

AgrC activity is likely influenced by other poorly defined factors [113,120]. Recently, a putative metalloprotease, MroQ, was shown to be required for Agr activity [126,127]. MroQ is proposed to function at the level of the peptide-processing module (AgrBD) [127] or via interaction with AgrC [126].

## 14. KdpDE

KdpDE TCSs are conserved in many pathogens and widely studied for their regulatory role in potassium (K^+^) transport and virulence gene expression [128]. In *S. aureus*, KdpDE activates the expression of the high-affinity potassium uptake system KdpABC and several virulence factors, such as the capsular biosynthesis gene cluster [129,130,131,132]. *kdpDE* is localized next to *kdpABC* but transcribed from a different promoter. KdpDE contributes to survival in human blood or in the presence of professional phagocytes [129]. In addition, KdpDE activates the expression of *sigS*, which encodes an alternative sigma factor [133]. KdpDE expression is increased by osmotic stress caused by NaCl or sucrose but decreased by K^+^. Transcriptional regulation of *kdpDE* expression seems not to be autoregulated since it occurs independently of the response regulator KdpE [129]. KdpDE expression is also positively regulated by Agr through the transcription factor Rot [129].

KdpD HK directly binds the second messenger molecule c-di-AMP through its intracellular universal stress protein domain [134]. A conserved SXS-X20-FTAXY motif is important for this binding, and c-di-AMP binding inhibits KdpDE signaling. It is proposed that under salt stress conditions, c-di-AMP inhibition is relieved, leading to KdpD activation. Of note, some MRSA strains harboring staphylococcal cassette chromosome *mec* type II (SCCmecII) contain a second *kdp* operon [135].

## 15. HssRS

The HssRS (heme sensor) system senses the level of extracellular hemin and activates the expression of *hrtAB*, which encodes a heme-regulated efflux pump, thereby sustaining heme homeostasis in *S. aureus* [136,137]. Genomics-based approaches have not revealed a potential heme-binding domain in HssS [136]. It was hypothesized that HssS might sense any toxic effect caused by heme [137]. The system is important to efficiently protect *S. aureus* from the toxic effects of heme.

## 16. NreBC

NreBC (nitrogen regulation) is important for nitrate and nitrite reduction [138]. The system is part of the *narGYJI-nreABC* operon that encodes nitrate reductase and the GAF (cGMP-specific phosphodiesterases, adenylyl cyclases, and FhlA)-domain-containing NO_3_^−^ sensor NreA and is best studied in the nonpathogenic *Staphylococcus carnosus* [139,140]. NreB is a bifunctional HK with integral cryptic phosphatase activity. Activation of phosphatase activity and dephosphorylation of RR, NreC-P requires NreA as a cofactor [140]. In the presence of NO_3_^−^, the NreA-NO_3_^−^ complex shows decreased interaction with NreB, allowing NreB phosphorylation. NreB is the only *S.* *aureus* HK with a cytoplasmic sensor kinase. NreB is an oxygen-sensing protein containing a Fe-S cluster at the N-terminus. Under anoxic conditions, NreB contains an oxygen-sensitive [4Fe-4S]^2+^ cluster and is phosphorylated at the conserved histidine residue. Disassembly of the [4Fe-4S]^2+^ cluster to a [2Fe-2S]^2+^ cluster upon exposure to oxygen leads to decreased autophosphorylation of NreB [139]. Thus, under anoxic conditions, NreB with the [4Fe-4S]^2+^ cluster exists in the active state, but when no NO_3_^−^ is available, it remains inhibited by NreA.

## 17. BraRS

The BraRS (bacitracin resistance-associated) system [141], also called NsaRS (nisin susceptibility associated) [142], is essential for bacitracin and nisin resistance in *S. aureus*. The sensor kinase BraS is classified as an IM-HK and harbors an extremely short extracellular loop of only three amino acids [141]. The ABC transporters BraDE and VraDE are involved in bacitracin resistance, and their operon expression is in turn induced by bacitracin and nisin through *braRS*. BraDE is important for the detection of antibiotics, whereas VraDE confers resistance by functioning as an efflux pump. Bacitracin is sensed by BraDE, and the signal is transduced to BraRS through a mechanism that remains to be elucidated, but that likely involves interaction between BraE and BraS [141]. Nisin resistance can be selected by *braRS* mutations, including SNPs in the promoter region of *braRS.* This results in constitutive induction of VraDE expression even in the presence of the unphosphorylated form of the BraR mutant protein [143]. Although the system is only involved in bacitracin and nisin resistance, other antimicrobials targeting the cell wall also activate its expression [144]. Thus, BraRS is involved in sensing disruptions of the cell envelope of *S. aureus.* Significant homologies have been found between the *braRS* and *graSR* systems in various staphylococcal species. Interestingly, *bsaRS* homologues are mainly present in skin-colonizing staphylococci [145].

## 18. Conclusions and Outlook

The synthesis of many factors involved in virulence and stress resistance is controlled by TCSs. TCSs allow the bacteria to properly adapt to the diverse stresses encountered during infection. The mechanisms by which HKs sense are only partially understood. Structural and biochemical analyses of purified *S. aureus* HKs are still rare, largely because most of the HKs are membrane proteins that are difficult to purify. HK phosphorylation activity in living bacteria cannot be measured directly and is often implied from gene expression analysis of selected target genes. However, the expression of many target genes is controlled by various regulators [7], which often impedes the interpretation of the data. Thus, the development of well-validated sensors based on confirmed or modified target genes would significantly improve the available experimental tools. Such genes should primarily be responsive to a single RR with an approved RR binding motif. To allow better readout, additional motifs in the selected promoter regions can be modified to improve the specificity of synthetic biosensors. In vitro assays to analyze signaling mechanisms in reconstituted liposomes and their use in structural studies are needed to gain further insights into the mechanisms underlying signal transduction.

An improved understanding of HK function can guide screens to search for new TCS inhibitors. TCSs are attractive candidate targets in the development of new antimicrobials. Specific inhibitors are assumed to have few undesirable side effects in mammals and might be used to specifically block virulence and/or resistance. Such blockers are thought to be less prone to the development of resistance because bacterial growth is usually not inhibited. Non-native small molecules and peptides capable of interfering with TCSs have been analyzed and have shown significant value as research tools and as potential antivirulence agents [146]. “Quorum quenchers” (inhibitors of quorum sensing) are of special interest and have been shown to block *S. aureus* virulence in animal models [115,147,148,149]. However, they may also promote biofilm formation, which has to be considered before clinical application. Inhibitors of other *S. aureus* TCSs, such as VraS [150], SaeS [149], or WalK [151], are also under investigation.

## Figures and Tables

**Table 1:** Genetic organization, regulation, and function of *S. aureus* TCSs.

HK	Annotation in USA300	Genetic Organization ^1^	Autoregulation, Regulation by Other Regulators ^2^	Prototypic, Verified Target Genes	Signal	Function
WalK	SAUSA300_0021	*→walR**K**HI*	auto	*atlA, sceD*	unknown	Cell division, autolysis
HptS	SAUSA300_0218	*hptA**S**R* *←→uhpT*	CcpA neg	*uhpT*	Glucose-6-P	Glucose-6-P import
LytS	SAUSA300_0254	*→lyt**S**R* *→lrgAB*	auto, SpoVG	*lrgA*	Membrane potential	Cell wall metabolism
GraS	SAUSA300_0646	*→graXR**S*** *→vraFG*	no auto	*vraF, mprF*	Indolicidin, Mellitin, Nisin, LL-37, Colistin, Polymyxin B	Surface charge CAMP Resistance
SaeS	SAUSA300_0690	*→saePQR**S*** *→saeRS*	auto, CodY neg, Agr, Rot neg, auto neg	*saeP, coa, eap*	HNP1–3	Virulence
TCS-7 (DesK)	SAUSA300_1219	*→SauSa300_1217-* *SauSa300_1218-des**K**R*		*SauSa300_1217*	Temperature	
ArlS	SAUSA300_1307	*→arlR**S***	SigB	*mgrA*	Low Manganese, Low glucose	Cell wall surface proteins, Manganese homeostasis
SrrB	SAUSA300_1441	*→srrA**B***	Rex	*qox, nrdG, cydA, scdA*	Reduced menaquinone (MQ-H_2_)	Oxidative stress
PhoR	SAUSA300_1638	*→phoP**R***		*pstSCAB*	Low P_i_	Phosphate homeostasis
AirS	SAUSA300_1799	*→air**S**R*		*crtO*	Oxygen	Redox sensing
VraS	SAUSA300_1866	*→vraUT**S**R*	auto	*relP, vraX*	Cell wall damage	Cell wall stress resistance
AgrC	SAUSA300_1991	*RNAIII* *←→agrBD**C**A*	auto, CodY neg, SarA, sRNAs	*RNAIII, psm*	Autoinducing peptide (quorum sensing)	Virulence
KdpD	SAUSA300_2035	*kdpABC* *←→kdp**D**E*	Rot neg	*kdpABC*	c-di-AMP	K^+^ homeostasis
HssS	SAUSA300_2309	*hrtAB* *←→hssR**S***		*hrtAB*	Hemin toxicity	Heme detoxification
NreB	SAUSA300_2338	*→nreGYJI* *→nreA**B**C*	auto, Rex	*nreG*	Low oxygen, High NO^3^-	Nitrogen respiration
BraS	SAUSA300_2558	*→bra**S**R* *→braDE*		*vraDE*	Bacitracin, Nisin	Bacteriocin resistance
TCS-2	SA0067	*kdpABC←→kdp**D**E* homologue on Mec Island				

^1^ Bold font is used to indicate the gene coding for HK. Arrows indicate orientation of transcription. ^2^ auto indicates autoregulation, interactions that result in inhibition are indicated with “neg”.

## Data Availability

Not applicable.
